# Army Appointments

**Published:** 1858-09

**Authors:** 


ARMY APPOINTMENTS.—The Army Medical Board met at Richmond, in April last, and selected but two of twenty-seven candidates who were examined. Drs. J. H. Bill, of Pa., and J. H. Berrien, of Ga., were the successful candidates.

